# The Antibacterial and Larvicidal Potential of Bis-(2-Ethylhexyl) Phthalate from *Lactiplantibacillus plantarum*

**DOI:** 10.3390/molecules27217220

**Published:** 2022-10-25

**Authors:** Muhammad Rizwan Javed, Mahwish Salman, Anam Tariq, Abdul Tawab, Muhammad Kashif Zahoor, Shazia Naheed, Misbah Shahid, Anam Ijaz, Hazrat Ali

**Affiliations:** 1 Department of Bioinformatics and Biotechnology, Government College University Faisalabad (GCUF), Allama Iqbal Road, Faisalabad 38000, Pakistan; 2 Department of Biochemistry, Government College University Faisalabad (GCUF), Jhang Road, Faisalabad 38000, Pakistan; 3 National Institute for Biotechnology and Genetic Engineering (NIBGE), Jhang Road, Faisalabad 38000, Pakistan; 4 Department of Zoology, Government College University Faisalabad (GCUF), Allama Iqbal Road, Faisalabad 38000, Pakistan; 5 Department of Chemistry, Government College University Faisalabad (GCUF), Jhang Road, Faisalabad 38000, Pakistan

**Keywords:** *Lactiplantibacillus plantarum* BCH-1, Bis-(2-ethylhexyl) phthalate, antibacterial activity, mosquito larvicidal activity

## Abstract

Lactic acid bacteria produce a variety of antibacterial and larvicidal metabolites, which could be used to cure diseases caused by pathogenic bacteria and to efficiently overcome issues regarding insecticide resistance. In the current study, the antibacterial and larvicidal potential of Bis-(2-ethylhexyl) phthalate isolated from *Lactiplantibacillus plantarum* BCH-1 has been evaluated. Bioactive compounds were extracted by ethyl acetate and were fractionated by gradient column chromatography from crude extract. Based on FT-IR analysis followed by GC-MS and ESI-MS/MS, the active compound was identified to be Bis-(2-ethylhexyl) phthalate. Antibacterial potential was evaluated by disk diffusion against *E. coli* (12.33 ± 0.56 mm inhibition zone) and *S. aureus* (5.66 ± 1.00 mm inhibition zone). Larvicidal potency was performed against *Culex quinquefasciatus* Say larvae, where Bis-(2-ethylhexyl) phthalate showed 100% mortality at 250 ppm after 72 h with LC_50_ of 67.03 ppm. Furthermore, after 72 h the acetylcholinesterase inhibition was observed as 29.00, 40.33, 53.00, 64.00, and 75.33 (%) at 50, 100, 150, 200, and 250 ppm, respectively. In comet assay, mean comet tail length (14.18 ± 0.28 μm), tail DNA percent damage (18.23 ± 0.06%), tail movement (14.68 ± 0.56 µm), comet length (20.62 ± 0.64 µm), head length (23.75 ± 0.27 µm), and head DNA percentage (39.19 ± 0.92%) were observed at 250 ppm as compared to the control. The current study for the first time describes the promising antibacterial and larvicidal potential of Bis-(2-ethylhexyl) phthalate from *Lactiplantibacillus plantarum* that would have potential pharmaceutical applications.

## 1. Introduction

The development of resistance in pathogenic micro-organisms, such as Gram-positive vancomycin-resistant *Staphylococcus aureus* (VRSA) and methicillin-resistant *S. aureus* (MRSA), have established a great interest in the discovery of natural products for the control of drug-resistant pathogens and new diseases [1]. In addition, there is a lack of potential treatment especially for extensively drug-resistant Gram-negative pathogenic micro-organisms, such as *Klebsiella pneumoniae* and *Escherichia coli* [2]. Moreover, many antibiotics are sometimes associated with adverse effects on the host (hypersensitivity, allergic reaction, and immunosuppression) [3]. In addition, mosquitoes are one of the most medically significant vectors, and they transmit parasites and pathogens, which continue to have a devastating impact on human beings and other animals [4]. Several mosquito species of *Culex, Anopheles,* and *Aedes* genera are pathogenic vectors of many vector-borne diseases affecting animal and human health, such as malaria, dengue, yellow fever, chikungunya, Japanese encephalitis, and filariasis [5,6]. *Culex quinquefasciatus* (Say), a brown colored and medium-sized mosquito is the primary vector of *Wuchereria bancrofti*, a nematode that causes lymphatic filariasis in Asia and infects more than 100 million people worldwide [7]. Some previous studies have also reported that the female *C. quinquefasciatus* mosquito is a causative agent of zika virus [8,9]. Many synthetic insecticides, such as organophosphates (fenthion and temephos), are used to control biological vectors, but their use as an insecticide has been prohibited due to associated health risks [10]. Moreover, the continuous use of synthetic insecticides results in resistance development within mosquitoes [11]. Therefore, the development of eco-friendly and target-specific antibacterial and larvicidal agents for the control of pathogens and mosquito larvae is of primary importance [12].

Natural bioactive microbial metabolites exhibiting unusual structures have had a significant role in drug discovery over the last few decades. Secondary metabolites from micro-organisms are considered as a good alternative to synthetic drugs due to their non-hazardous and bio-friendly behavior [13]. After 50 years of intensive research on secondary metabolites from micro-organisms, studies on natural bioactive compounds are still an emerging research topic. Various species of lactic acid bacteria (LAB) and their metabolites have potent antibacterial activity against pathogenic bacteria, such as *Salmonella* sp., *E. coli*, *Listeria monocytogenes*, *S. aureus*, *Bacillus cereus*, *Bacillus megaterium*, and *Pseudomonas aeruginosa* [14,15]. LAB are also exhibiting inhibitory potential against pathogenic fungi, namely, *Aspergillus flavus* and *Aspergillus fumigatus* [16]. Some LAB species, such as *Enterococcus durans, Lactobacillus plantarum*, and *Lactobacillus johnsonii*, also have interesting applications for biocontrol of disease-causing biological vectors, viz, *C. quinquefasciatus* (Mosquitoes), *Drosophila melanogaster* (Common fruit fly), and *Musca domestica* (Housefly), respectively [17,18,19]. *Lactiplantibacillus plantarum* (formerly known as *Lactobacillus* *plantarum*) [20] is a potent LAB specie that is evaluated in the current study. It produces multiple bioactive secondary metabolites having a variety of structures, such as organic acids, fatty acids, hydrogen peroxide, bacteriocins, and reuterin. Such bioactive metabolites exhibit diversified biological activities [21]. 

Phthalates are esters of phthalic acid, also called as esters of benzene-1,2-dicarboxylic acid, having a benzene ring with two ester groups. Bis-(2-ethylhexyl) phthalate (BEHP) has been reported as a potent bioactive secondary metabolite, naturally produced by bacterial, fungal, and algal species [22,23,24]. Precisely, several species of bacteria: *Bacillus subtilis* AD35, *Bacillus cereus* [25,26]; fungi: *Aspergillus awamori*, *Epicoccum nigrum* [27,28]; and algae: *Bangia atropurpurea* [22] have yet been reported to produce BEHP and other phthalates, such as metabolites, e.g., dibutyl phthalate (DBP) [22,29]. These metabolites play an important role in many areas [30]. El-Sayed et al. [31] reported BEHP production with cytotoxic and antimicrobial potential by *Streptomyces mirabilis.* Rajamanikyam et al. [32] reported the larvicidal and antibacterial activities of BEHP isolated from *Brevibacterium mcbrellneri* bacterium. So, the focus of the current study is to purify bioactive metabolite Bis-(2-ethylhexyl) phthalate (BEHP) from *Lactiplantibacillus plantarum* BCH-1 and to analyze its structural elucidation by Fourier Transform Infrared (FT-IR) Spectroscopy, Gas Chromatography-Mass Spectrometry (GC-MS), and Electrospray Ionization Mass Spectrometry (ESI-MS/MS). The purified metabolite was also evaluated for its antibacterial activity and larvicidal potential. To the best of our knowledge, *Lactiplantibacillus* *plantarum* has not been reported as a source of BEHP. Moreover, the antilarval activity against *Culex quinquefasciatus*, acetylcholinesterase enzyme (AChE) inhibitory activity and effects on DNA damage have also been discussed.

## 2. Results

### 2.1. Antibacterial Assay of Crude and Purified Metabolites 

**Figure 1:**
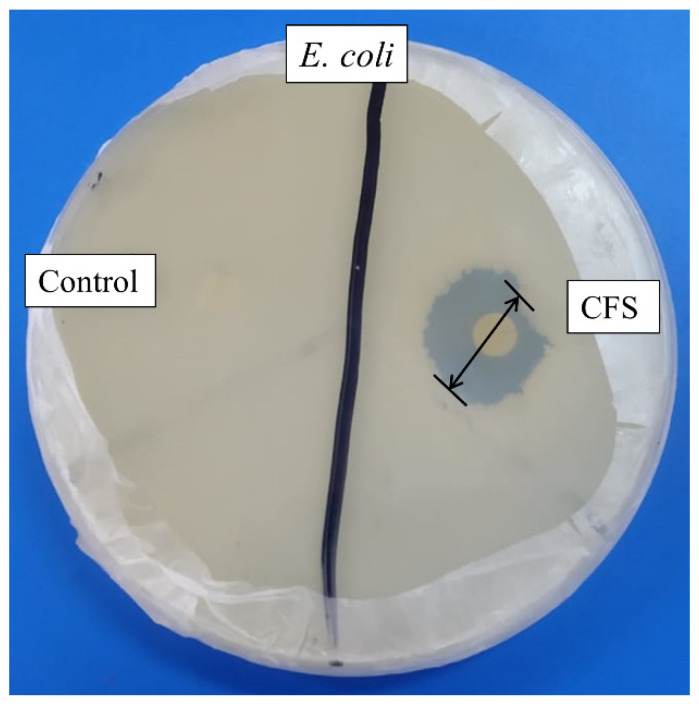
Antibacterial activity of cell-free supernatant (CFS) against *E. coli*. The MRS broth was used as negative control, while the disk diameter was 6 mm.

**Figure 2:**
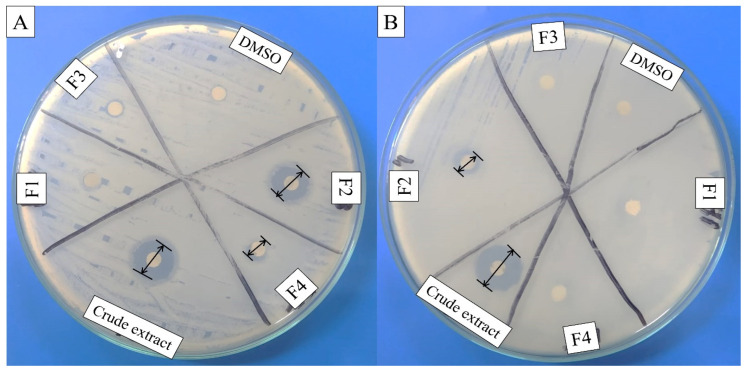
Antibacterial activity of ethyl acetate crude extract and column purified fractions (F1, F2, F3, and F4) against (**A**) *E. coli* (Gram-negative) and (**B**) *S. aureus* (Gram-positive). The DMSO (organic solvent) was used as negative control, while the disk diameter was 6 mm.

Cell-free supernatant (CFS) of 72 h culture of *Lactiplantibacillus* *plantarum* BCH-1 showed the presence of bioactive metabolites by exhibiting antibacterial activity (inhibition zone of 16.00 ± 1.00 mm) against *E. coli* (Figure 1). The ethyl acetate crude extract also showed potent antibacterial activity (inhibition zones of 14.00 ± 1.00 mm and 13.66 ± 1.52 mm) against *E. coli* and *S. aureus*, respectively (Figure 2). When ethyl acetate crude extract was passed through the column, a total of four purified fractions (F1, F2, F3, and F4) were collected by column chromatography. Of these fractions, F2 (Figure 2) showed stronger antibacterial activity (inhibition zones 12.33 ± 0.56 mm and 5.66 ± 1.00 mm) against *E. coli* and *S. aureus*, respectively, as compared to other collected fractions, F1 = ~0.00 mm and F3 = ~0.00 mm against both pathogenic bacterial strains, whereas F4 showed inhibition zones of 1.66 ± 1.00 mm and ~0.00 mm against *E. coli* and *S. aureus*, respectively. The DMSO was used as a negative control, which showed no activity (Figure 2). The column chromatographic mobile phase solvent details have been mentioned in Table 1. These results suggested that various types of bioactive metabolites were present in CFS and not all metabolites were biologically active. The F2 fraction had maximum bioactivity and therefore this fraction was selected for further analysis. 

### 2.2. Structural Determination of Bioactive Metabolite

#### 2.2.1. FT-IR Analysis

**Figure 3:**
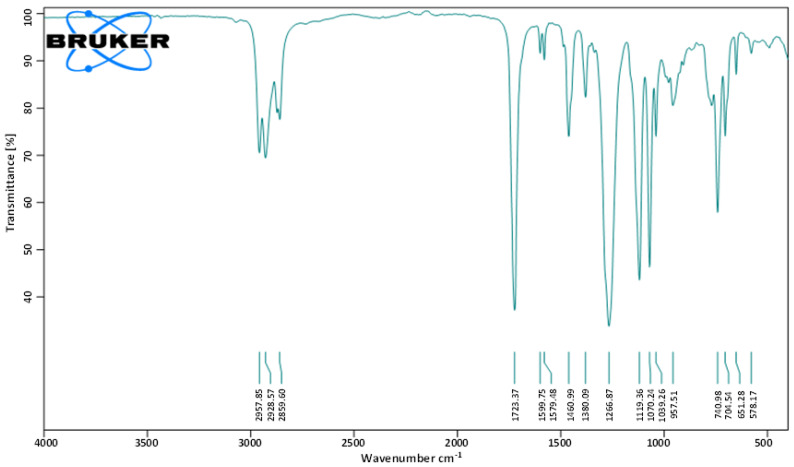
FT-IR spectrum of the bioactive fraction (F2) of ethyl acetate crude extract from *L.* *plantarum* BCH-1.

To identify the molecular functional groups of the bioactive compounds in the fraction F2, it was subjected to FT-IR analysis. Various peaks were recorded. The peaks observed at 1039.26 and 1070.24 cm^−1^ show the presence of the C-O group, while 1723.37 cm^−1^ corresponds to the presence of the C=O bond. The peaks at 1460.99 and 1599.75 cm^−1^ reflect the presence of the C=C aromatic group, while the peak at 2859.60 cm^−1^ indicates the characteristic of the C-H alkane group. Hence, the observed spectral data suggested the presence of aromatic ester in the isolated metabolite (Figure 3).

#### 2.2.2. GC-MS Analysis

**Figure 4:**
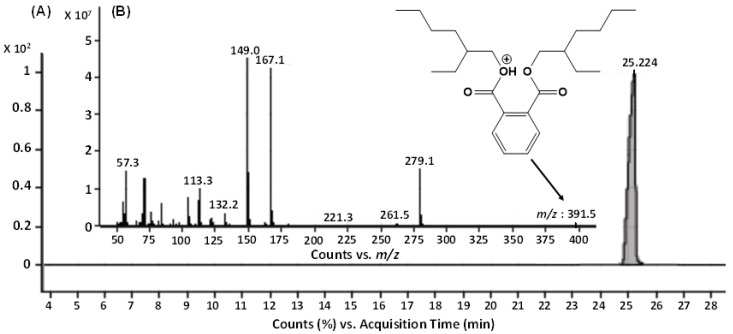
GC-MS analysis of the bioactive fraction (F2) of ethyl acetate crude extract from *L.* *plantarum* BCH-1. (**A**) Total Ion Chromatogram (TIC) of F2 fraction (**B**) Mass spectrum of ion peak at 25.224 min.

**Figure 5:**
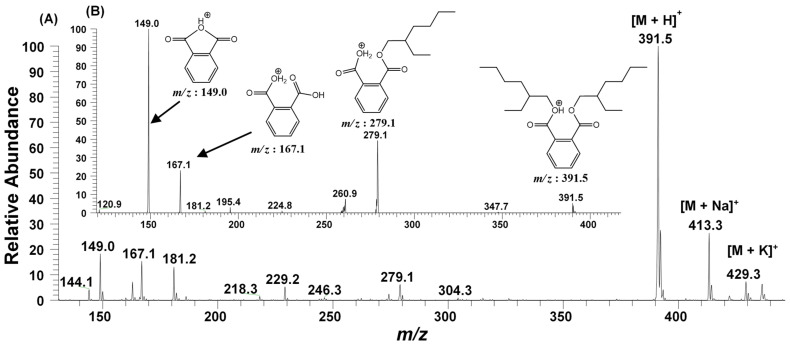
ESI-MS/MS of the fraction (F2), having bioactive metabolite (BEHP), (**A**) Full MS scan of BEHP (**B**) MS^2^ of the ion peak at *m*/*z* 391.5.

The GC-MS analysis of the bioactive fraction (F2), extracted in ethyl acetate, resulted a single major peak at the time interval of 25.224 min, indicating a purified product (Figure 4A). The mass spectrum of this moiety was recorded with the molecular ion peak [M+H]^+^ at *m*/*z* 391.5. The other daughter ion peaks at *m*/*z* 113, 167, 279 and the base peak at *m*/*z* 149 were also observed. Using the library search and studying the molecular fragmentation, this metabolite was identified as Bis-(2-ethylhexyl) phthalate (BEHP) (Figure 4B). For confirmation, this moiety was further subjected to ESI-MS/MS (Figure 5).

#### 2.2.3. ESI-MS/MS Analysis

In accordance with the GC-MS results (Figure 4), the ESI-MS/MS of F2 fraction in full scan at positive ionization mode also generated the molecular ion peak at *m/z* 391.5 [M + H]^+^ along with their sodiated ionic peak at *m*/*z* 413.3 [M + Na]^+^ and potassium adducts at *m*/*z* 429.3 [M + K]^+^. Additionally, several other daughter ionic peaks, e.g., *m*/*z* 279.1, 181.2, 167.1, and 149.0 were also obtained (Figure 5A). To elucidate its molecular structure, the molecular ion peak at *m*/*z* 391.5 was further subjected to MS^2^ fragmentation. The MS^2^ of this ion peak generated the daughter ion peaks at *m*/*z* 279.1, 167.1 and a base peak at *m*/*z* 149.0 along with a few other minor peaks. The elimination of the alkyl group from the ester linkage of carboxylic oxygen of BEHP resulted a mono-alkylated adduct with *m*/*z* 279.1. Similarly, the elimination of both the alkyl groups generated an ion peak at *m*/*z* 167.1. The removal of one alkyl group, along with the oxygen atom of ester linkage, generated a bicyclic adduct, with a base peak at *m*/*z* 149.0 (Figure 5B). The ESI-MS/MS analysis further supported the GC-MS results, suggesting the metabolite present in the F2 fraction to be the BEHP moiety.

### 2.3. Mortality of Culex quinquefasciatus Larvae

The BEHP concentration of 250 ppm showed the highest mortality (100%) after 72 h of exposure, while 61% and 78% mortality were noted after 24 and 48 h, respectively (Table 2). The mortality at 100 ppm of BEHP was observed as 75%, 61%, and 33% after 72, 48, and 24 h exposure, respectively, while 50 ppm concentration of BEHP exerts 61%, 43% and 23% mortality after 72, 48, and 24 h exposure, respectively. To exclude the mortality due to environmental factors, such as temperature, humidity, air, and light, a control group was also evaluated under similar conditions (Table 2). The results suggested that the antilarval activity of BEHP against *C. quinquefasciatus* larvae was concentration and time dependent. Moreover, the values of lethal concentration (LC_50_) suggested that BEHP showed the highest toxicity with low LC_50_, which decreases with time (Table 3). The LC_50_ was 67.03 ppm after 72 h exposure and 108.66 ppm and 186.11 ppm after 48 and 24 h, respectively.

### 2.4. Acetylcholinesterase (AChE) Enzyme Inhibitory Activity

The effect of BEHP on AChE in *C. quinquefasciatus* larvae was determined at different concentrations after 72 h (Table 4). The highest inhibitory activity (75.33%) was observed at the highest concentration (250 ppm) of BEHP. The concentrations of 50, 100, 150, 200 ppm with their maximum inhibitory activity were also evaluated (Table 4). The enzyme inhibitory assay showed that enzyme inhibition was increased with increasing the BEHP concentration. 

### 2.5. Comet Assay 

**Figure 6:**
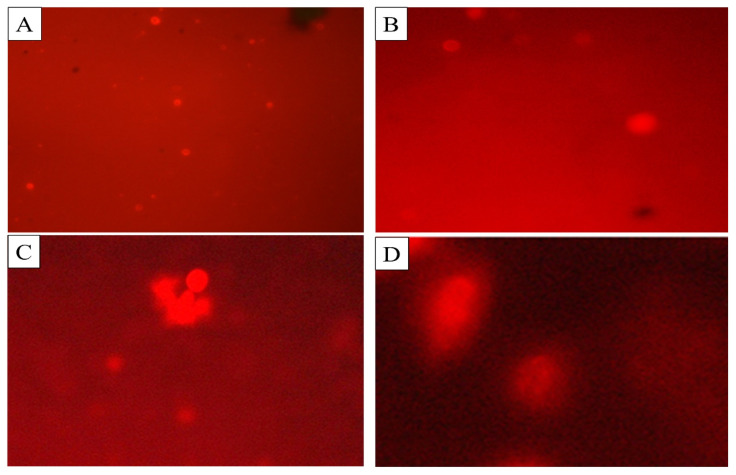
DNA damage of *C. quinquefasciatus* larvae at different concentrations of BEHP determined through Comet assay. (**A**): Control (without BEHP), (**B**): 100 ppm concentration, (**C**): 150 ppm concentration and (**D**): 250 ppm concentration of BEHP.

The comet assay was performed to determine the DNA damage in the cells of *C. quinquefasciatus* larvae treated with BEHP. Three different concentrations (100 ppm, 150 ppm, and 250 ppm) with control were used to observe DNA fragments migration by agarose gel-electrophoreses (Table 5; Figure 6). The highest mean value of tail length (14.18 ± 0.28 μm), tail DNA% damage (18.23 ± 0.06%), tail movement (14.68 ± 0.56 μm), comet length (20.62 ± 0.64 µm), head length (23.75 ± 0.27 µm) and head DNA % (39.19 ± 0.92%) were observed at 250 ppm concentration of BEHP. 

## 3. Discussion

The finding of new biomolecules from micro-organisms are not an ending process to fulfill the eternal need for novel potent metabolites for combating pathogens [33]. In the current study, *L.* *plantarum* BCH-1 CFS showed antibacterial potential against *E. coli* (Figure 1) and bioactive metabolites from CFS were extracted by ethyl acetate because ethyl acetate was found to be an efficient organic solvent for extracting the bioactive metabolites as compared to dichloromethane and n-hexane [16]. The same solvent has already been reported, such as for the extraction of bioactive metabolites produced by *Bacillus subtilis* [26]. In the current study, ethyl acetate crude extract showed significant antibacterial activity against *E. coli* and *S. aureus* (Figure 2). The purification of these bioactive metabolites was carried out using column chromatography. A total of four fractions were evaluated for antibacterial potential determination among which the F2 fraction showed significant antibacterial activity against *E. coli* and *S. aureus* (Figure 2). 

The active metabolite within the fraction F2 was chemically characterized by FT-IR (Figure 3). Its molecular structure elucidation was carried out using GC-MS (Figure 4). The metabolite was found to be Bis-(2-ethylhexyl) phthalate (BEHP). The ESI-MS/MS analysis (Figure 5) also supported the findings. The results of spectral data were correlated with previous findings in the literature [25,31,34,35]. This metabolite is also known as Di-(2-ethylhexyl) phthalate (DEHP) and Bis (2 ethyl hexyl) Benzene-1,2-dicarboxylate. Additionally, reports also suggest that BEHP has antitumor [23] and antileukemic [36] effects. BEHP was first isolated from the microbial source of *Streptomyces* sp. [37] and has been reported as bioactive metabolite from many bacterial species, e.g., *B. subtilis* [26], *Pseudomonas* sp. PBO1 [38]. Anju et al. [25] had reported the identification of BEHP as one of the metabolites produced by a nematode symbiotic bacterium associated with a novel entomopathogenic nematode *Rhabditis* species. Previously, authors have reported that *L. plantarum* also produce such types of metabolites, e.g., diisooctyl phthalate [39]. However, no specific results have been reported on the isolation of Bis-(2-ethylhexyl) phthalate (BEHP); hence, the current report describes the isolation and characterization of BEHP from *L. plantarum* BCH-1 for the first time.

In addition, the BEHP isolated from *L.* *plantarum* BCH-1 was analyzed for larvicidal activity and showed the highest mortality in *C. quinquefasciatus* larvae after 72 h of exposure. The mortality showed direct proportionality with increasing the exposure time and dose concentrations (Table 2). Torres et al., [17] had reported that organic acids produced by *L. johnsonii* CRL 1647, exert pupicidal and larvicidal effects on *M. domestica*. Gupta et al. [18] had also reported in vivo evaluation of toxic effect of antimicrobial peptides isolated from *L. plantarum* LR/14 on *D. melanogaster*, DNA fragmentation, and premature apoptosis, confirming that the peptides have a dose-dependent toxic property. Moreover, BEHP also showed AChE inhibition activity against mosquito larvae *C. quinquefasciatus* (Table 4). AChE is a serine hydrolase enzyme present at neuromuscular junctions and cholinergic brain synapses. Its function is impulse transmission termination at cholinergic synapses by breaking of neurotransmitter acetylcholine (Ach) to choline and acetate. AChE inhibitors inhibit cholinesterase enzyme from breaking down Ach [40]. It was reported that AChE is a sensitive enzyme affected by insecticides [41]. Previously, researchers studied the effect of BEHP on AChE inhibition against *Aedes aegypti* larva and they observed significant results of the BEHP effect on AChE inhibition [32]. Ganesan et al. also studied the BEHP effect on AChE activity in *C. quinquefasciatus* larvae that was observed to be dose dependent [42]. Furthermore, in the current study the BEHP also showed DNA damage (Table 5 and Figure 6) in *C. quinquefasciatus*, suggesting that BEHP could bind with the DNA of organisms. The binding could affect the replication process, thereby causing the death of susceptible organisms [35,43] through the mechanisms not studied yet. The observed mortality of *C. quinquefasciatus* larvae can be caused by the modulate AChE enzymatic activity and can also be attributed due to DNA damage [44]. 

## 4. Materials and Methods

### 4.1. Reagents and Chemicals 

The de Man, Rogosa & Sharpe (MRS) broth/agar, Glycerol (Analytical grade), Nutrient agar/broth, HPLC grade organic solvents (Ethyl acetate, n-hexane, Chloroform, Methanol, Dimethyl sulfoxide (DMSO), Ethanol), Sodium phosphate, Acetylcholine chloride, Fast blue B salt, Ethylenediaminetetraacetic acid (EDTA), Sodium chloride (NaCl), Tris, Triton X-100, Na_2_EDTA, Sodium hydroxide (NaOH) and 4′,6-diamidino-2-phenylindole (DAPI) were mainly purchased from Sigma-Aldrich, St. Louis, MO, USA. Silica gel (70–230 mesh) and Thin-layer chromatography (TLC) silica gel 60 F₂₅₄, aluminum sheet 20 × 20 cm^2^ were purchased from Merck, Darmstadt, Germany. 

### 4.2. Bacterial Strains and Growth Conditions

*Lactiplantibacillusplantarum* BCH-1 (KX388380) strain previously isolated from fermented rice rinsed water with lactose as a carbon source was cultured on MRS agar/broth at 37 °C [16] and was preserved at −80 °C in 15% (*v*/*v*) glycerol. The antibacterial potential was evaluated against Gram-positive *Staphylococcus aureus* and Gram-negative *Escherichia coli.* Bacterial cultures of test strains were obtained from Industrial Biotechnology Lab, Department of Bioinformatics and Biotechnology, GCUF, Pakistan and stocks were maintained on nutrient broth/agar, then stored at 4 °C. 

### 4.3. Collection and Rearing of Culex quinquefasciatus

Larvae and adult mosquitoes were collected from Bismillah Park of Ghulam Muhammadabad, Faisalabad, Pakistan after rainfall. The larvae samples were transferred into a beaker having water. The observed morphological features of mosquito larvae were: make an angle with the water surface, slender, short breathing tube, and blunt gills. These features confirmed them as *Culex quinquefasciatus* [45]. The identified larvae were brought to the Entomology Lab, Department of Zoology, GCUF, Pakistan for rearing (in enamel trays) under 12:12 (light:dark) photoperiod at 26 ± 1 °C and 60 ± 10% relative humidity (RH). The newly developed larvae were fed on ground fish food, while larvae that were 5 to 8 days old were fed daily on Purina cat food tablets. The pupae were kept in a tray and shifted into a rearing cage. After the development of adults from the pupa in 2–3 days, the male adults were fed with a 10% sucrose solution soaked in cotton in the beaker and kept in a cage. Meanwhile, the female adults were blood-fed for the development of oviposition by keeping a rat in the cage [11].

### 4.4. Fermentation Production of Secondary Metabolites 

Fermentation of *Lactiplantibacillus* *plantarum* BCH-1 (6L) culture was carried out in a fermenter (BioFer-010, ICCC, Islamabad, Pakistan) containing MRS broth medium (pH 6.4 ± 0.2) on constant stirring at 120 rpm for 72 h at 37 °C. After incubation, the cell-free supernatant (CFS) was prepared by centrifugation at 6000 rpm (4430× *g*) (Z326K, Hermle, Wehingen, Germany) for 10 min at 4 °C and filtered with 0.22 μm pore size disposable sterile filters (Advantec, Toyo Kaisha Ltd., Tokyo, Japan). The CFS was concentrated by Freeze-drying (Alpha 2-4 LSC basic, Christ, Osterode am Harz, Germany) [16] and was initially used to determine antibacterial potential.

### 4.5. Antibacterial Activity Assay

The antibacterial activity was carried out using disk diffusion method according to Atalla et al. [46] with slight modifications. The filter paper was punched to produce 6mm diameter paper disks, and these disks were autoclaved at 121 °C in a sealed bottle. The CFS was dissolved (30 mg/100 μL, *w*/*v*) in distilled water. Approximately 30 μL of CFS was applied on disks and left to dry at room temperature for a few minutes. The MRS broth was used as a negative control. Nutrient agar medium was prepared and the pathogenic bacterial strain (*E. coli*) (10^8^ CFU/mL) was spread over the plate using a sterilized cotton swab. The sample’s impregnated disks were carefully applied on inoculated nutrient agar plates by using sterilized forceps and incubated at 37 °C for 16 h under aerobic conditions. The antibacterial activity was determined as inhibition zones around the disk that were measured with a ruler in millimeters (mm). 

Antibacterial activity was determined by using the following formula [47]:

Inhibition Zone (mm) = Diameter of growth inhibition zone around the disk (mm) − Diameter of the disk (6 mm)

### 4.6. Extraction and Purification of Bioactive Metabolites 

Freeze-dried concentrated CFS was mixed in 50 mL sterile water and metabolites were extracted with ethyl acetate as extracting solvent (CFS:Ethyl acetate; 1:3 *v*/*v* ratio) according to Bukhari et al. [16]. The organic yellowish layer was collected, combined, and concentrated through rotavapor (R-210, Buchi, Flawil, Switzerland) under vacuum at a temperature below 40 °C. The crude extract obtained after the rotary evaporator was packed on a silica gel column (Chem glass CG-1196-18 Column Chromatography, 24/40 Outer Joint, 2000 mL Reservoir, 3in ID × 18 in length, 4 mm Stopcock) equilibrated with n-hexane. The gradient solvent system (100% n-hexane, gradient n-hexane:chloroform (85:15 to 15:85, *v*/*v*), 100% chloroform, gradient chloroform:ethyl acetate (95:5 to 5:95, *v*/*v*), 100% ethyl acetate and lastly with 100% methanol) was used in mobile phase with a gradual increase in polarity to elute the mixtures of compounds. The purity was determined by TLC under UV lamp (UVGL-58, Fullerton, CA, USA). The fraction presenting similar TLC spots were combined and again loaded to the column for isolation of single bioactive metabolite [48]. 

### 4.7. Antibacterial Activity of Crude Extract and Fractions 

The dried crude ethyl acetate extract was diluted [crude extract:water; 3:1 *v*/*v*)] in distilled water, while the dried collected fractions (F1, F2, F3 and F4) were redissolved (10 mg/100 μL, *w*/*v*) in DMSO (a polar aprotic solvent that dissolve both polar and nonpolar compounds) [49] for the antibacterial assay against test pathogenic bacterial strains (*E. coli* and *S. aureus*). The DMSO was used as negative control and the bioactivity was determined by following the previously described disk diffusion method [46,47]. After analysis, the fraction containing the bioactive compound was analyzed further as described in next sections. 

### 4.8. Structure Determination of Bioactive Metabolite

#### 4.8.1. Fourier Transform Infrared Spectroscopy (FT-IR)

In order to identify the molecular groups of bioactive fraction (F2), its FT-IR analysis (Tensor II, Bruker, Billerica, MA, USA) was conducted in the range of 4000 to 500 cm^−1^ [50]. The spectral data was plotted as wave number (cm^−1^) on x-axis versus %age transmittance at y-axis.

#### 4.8.2. Gas Chromatography-Mass Spectrometry (GC-MS)

The bioactive fraction (F2) was subjected to GC-MS analysis (Mass Hunter GC/MS TQ-7000, Agilent Technologies, Santa Clara, CA, USA) for its structural elucidation and identification. The oven temperature was set initially at 150 °C for 5 min and then increased to 300 °C with an increment of 10 °C per min and kept for 15 min. Helium was used as a carrier gas, with a flow rate of 14 mL/min [51]. The identification was based upon a 90% resemblance between MS spectra of unknown and reference compounds in MS spectral library (NIST14. LIB, Agilent Technologies, Santa Clara, CA, USA).

#### 4.8.3. Electrospray Ionization Mass Spectrometry (ESI-MS/MS) Analysis 

For further confirming the metabolite of F2 fraction and elucidating its molecular structure, the ESI-MS/MS (LTQ XL, Thermo Electron Corporation, Waltham, MA, USA) analysis was carried out by following the protocol described earlier [36] with a few minor modifications. Briefly, 2 mg of fraction F2 was dissolved in 1 mL of methanol: acetonitrile [80:20, *v*/*v*] mixture and run, using direct injection mode at 9 μL/min. The capillary temperature was set at 288 °C. The mass range was selected at *m*/*z* 100 to 1000 in positive ionization mode for data acquisition. The collision induced dissociation energy (CID) was manually selected in the range of 5 to 30 eV for obtaining favorable fragmentation. The sheath and auxiliary N_2_ gases were also adjusted manually. For the data analysis and structural elucidation, Xcalibur™ (version 3.0, Thermo Fisher Scientific, Waltham, MA, USA) and ChemDraw (version Chem Draw Ultra 8.0, PerkinElmer, Waltham, MA, USA) software were used.

### 4.9. Larvicidal Bioassay

Stock solution (1000 ppm; parts per million) was prepared by dissolving 100 mg of F2 fraction in 1 mL DMSO and the volume was raised to 100 mL with distilled water. From the stock solution, different dilutions of 50 ppm, 100 ppm, 150 ppm, 200 ppm and 250 ppm were prepared in 100 mL deionized water. The abovementioned concentrations of bioactive fraction (F2) having resultant compound were prepared to perform bioassay following the WHO protocol [52], while an untreated group (only DMSO in 100 mL water) was used to examine the effects of DMSO and environmental factors (air, light, temperature, and humidity) as a control. Twenty larvae (4th instar; fourth larval stage; L4) of *C. quinquefasciatus* were introduced to each concentration of the test compound in beakers containing 100 mL of water. The beakers were labeled properly and covered with muslin cloth. Each test was performed in triplicate with a control group and their mortality was recorded after 24, 48, and 72 h. The immovable dead larvae that showed no response with sharp pin probing were removed from beakers and stored in microcentrifuge tubes containing ethanol [11] to determine their Acetylcholinesterase (AChE) inhibition activity and DNA damage by comet assay.

### 4.10. Acetylcholinesterase Enzyme Assay

#### 4.10.1. Homogenate Preparation 

For Acetylcholinesterase enzyme (AChE) estimation, *C. quinquefasciatus* larvae were washed with distilled water and dried on blotting paper. After washing thoroughly, these larvae were homogenized by using cold sodium phosphate buffer (20 mM, pH 7.0) with Teflon hand homogenizer and subsequently centrifuged at 6000 rpm (4430× *g*) (Z326K, Hermle, Wehingen, Germany) for 20 min at 4 °C. The supernatant was used for the estimation of AChE. All glassware and solutions used for homogenization purpose, were placed at 4 °C before use and the prepared homogenate was also placed on ice until used for AChE estimation [44]. 

#### 4.10.2. Quantitative Determination of Acetylcholinesterase (AChE) Activity

For this purpose, 50 μL of enzyme solution was added to 50 μL of 2.6 mM acetylcholine chloride (as substrate) with 1 mL of sodium phosphate buffer (20 mM, pH 7.0) and incubated at 25 °C for 5 min. Finally, 400 μL of Fast blue B salt (0.3% *w*/*v*) was added to stop the reaction. The sample and control (without enzyme solution) were placed in a spectrophotometer (STA-8200V, Stalwart, Van Nuys, CA, USA) at 405 nm to observe optical density (OD) [41].

Enzyme inhibition percentage was calculated by the following formula:

$$\mathrm{Enzyme}\;\mathrm{inhibition}\;\%=\frac{\mathrm{OD}\;\mathrm{of}\;\mathrm{control}\;\mathrm{larvae}\;-\;\mathrm{OD}\;\mathrm{of}\;\mathrm{treated}\;\mathrm{larvae}}{\mathrm{OD}\;\mathrm{of}\;\mathrm{control}\;\mathrm{larvae}}\;\times \;100$$

### 4.11. Comet Assay 

Comet assay was performed for the determination of DNA damage [44] with slight modifications. For this purpose, the glass slide was prepared by coating with 150 μL of 1% (*w*/*v*) normal melting agarose (NMA). After drying, the stored sample of mosquito larvae suspended in 140 μL of 0.75% (*w*/*v*) low melting agarose (LMA) was layered over prepared glass slides (frosted ends) and left for drying at room temperature. Slides were covered with a coverslip (24 × 60 mm) and placed at 4 °C for 10 min to harden the agarose layer. After that, the coverslip was removed and slides were immersed in cold lysis solution (100 mM EDTA, 2.5 M NaCl, 10 mM Tris, 1% (*v*/*v*) Triton X-100, pH 10 and 5% (*v*/*v*) DMSO) at 4 °C for 2 h in dark. For DNA unwinding, slides were placed in an electrophoretic tank (horizontal) filled with cold electrophoretic buffer (300 mM NaOH, 1 mM Na_2_EDTA, pH 13) for 25 min and electrophoresis was performed in the same buffer for 20 min at 25 V and 300 mA (0.73 V/cm). After electrophoresis, the slides were stained with 20 μL of DAPI (1 μg/mL) per gel after gel neutralization by washing twice with 0.4 mM Tris (pH 7.5). The slides were scored under a fluorescence microscope (Olympus BX50, IMEB Inc., San Marcos, TX, USA) using Komet 5.5 Image Analysis System (Kinetic Imaging Ltd., Nottingham, UK). A total of 100 randomly selected (50 cells per 2 replicate slides) per treatment were scored [11,53].

### 4.12. Data Analysis

Zones of antibacterial activity inhibition were measured as mean ± standard deviation of three replicates (*n* = 3). For antilarval activity, Abbot’s formula [54] was applied for the analysis of mortality data. The mortality percentage of larvae data for different concentrations were subjected to the Probit analysis program by using Minitab software package (version 19.0, Minitab Ltd., Coventry, UK) to determine the LC_50._ Corrected mortality data were subjected to ANOVA using SPSS software package for Windows (Version 23.0, IBM Corporation, Armonk, NY, USA) and means were separated through Tukey’s HSD (Honest Significant Difference) test. The value of *p* ≤ 0.05 was considered statistically significant [36]. DNA damage in cells was assessed by following distinct types of measurements: (1) DNA comet tail length, (2) Fragmented DNA percentage (%) present in tail, (3) Tail movement, (4) Comet length, (5) Head length, and (6) Head DNA percentage (%) after electrophoresis [55]. 

## 5. Conclusions

Bioactive metabolite Bis-(2-ethylhexyl) phthalate (BEHP) was isolated from LAB species *Lactiplantibacillus* *plantarum* BCH-1, which exhibited potent antibacterial and larvicidal activity with significant acetylcholinesterase inhibition activity and DNA damage against *Culex quinquefasciatus* Say larvae. Furthermore, the identity of the resultant bioactive metabolite was confirmed using FT-IR, GC-MS, and ESI-MS/MS. More precisely, the bioactive metabolite, BEHP or other phthalate such as DBP could be used to combat disease-causing pathogenic micro-organisms and to control various biological vectors.

## Figures and Tables

**Table 1:** Details of the column chromatography and purified metabolite.

Sr. No.	Column Chromatography Mobile Phase Solvent of Bioactive Fraction	Metabolite Name	Molecular Formula	Molecular Mass
1	85% chloroform in n-hexane	Bis-(2-ethylhexyl) phthalate (BEHP)	C_24_H_38_O_4_	390.564 g/mol

**Table 2:** Mortality (%age) of 4th instar *C. quinquefasciatus* larvae at different time intervals and concentrations of BEHP.

Exposure Time	BEHP Concentration (ppm)					
	0 (E.F.)	50	100	150	200	250
	Mortality % Age (Mean ± S.E.)					
24 h F = 84.30; d.f. = 5; *p* < 0.05	0.00 ± 0.00 ^a^	23.33 ± 3.33 ^b^	33.33 ± 1.66 ^bc^	43.33 ± 3.33 ^c^	55.00 ± 2.88 ^d^	61.66 ± 1.66 ^d^
48 h F = 138.75; d.f. = 5; *p* < 0.05	0.00 ± 0.00 ^a^	43.33 ± 3.33 ^b^	61.66 ± 1.66 ^c^	71.66 ± 1.66 ^cd^	76.66 ± 1.66 ^d^	78.33 ± 4.40 ^d^
72 h F = 241.13; d.f. = 5; *p* < 0.05	0.00 ± 0.00 ^a^	61.66 ± 1.66 ^b^	75.00 ± 2.88 ^c^	88.33 ± 4.40 ^d^	91.66 ± 1.66 ^de^	100 ± 0.00 ^e^

Where ^a^, ^b^, ^c^, ^d^, ^e^, ^bc^, ^cd^, and ^de^ mean different letters within each treatment are statistically significant according to Tukey’s HSD test. F = ratio of between group variation and within group variation; d.f. = degree of freedom; *p* < 0.05 = statistically significant and E.F. = Environmental Factor group.

**Table 3:** Toxicity of BEHP against *C. quinquefasciatus* larvae.

Observation Interval (h)	N	LC_50_ (Lower ± Upper Value)	Slope ± S.E.	X^2^	*p*-Value
24	20	186.11 (169.86 ± 205.83)	0.0071684 ± 0.0007120	17.55	0.002
48	20	108.66 (94.43 ± 122.00)	0.0082078 ± 0.0007042	48.06	0.000
72	20	67.03 (56.82 ± 76.34)	0.0144255 ± 0.0010709	54.91	0.000

**Table 4:** Effect of different BEHP concentrations on percent inhibition of acetylcholinesterase (AChE) activity in *C. quinquefascitus* larvae after 72 h.

BEHP Concentration (ppm)				
50 ppm	100 ppm	150 ppm	200 ppm	250 ppm
AChE (% inhibition) F = 476.01; d.f. = 4; *p* < 0.05				
29.00 ± 0.57 ^a^	40.33 ± 0.88 ^b^	53.00 ± 1.15 ^c^	64.00 ± 0.57 ^d^	75.33 ± 0.88 ^e^

Where ^a^, ^b^, ^c^, ^d^, and ^e^ letters are showing statistically significant results according to Tukey’s HSD test. F = ratio of between group variation and within group variation; d.f. = degree of freedom and *p* < 0.05 = statistically significant.

**Table 5:** Comet parameters and DNA damage of *C. quinquefasciatus* larvae at different concentrations of BEHP.

Comet Parameters and DNA Damage	BEHP Concentration (ppm)			
	Control	100 ppm	150 ppm	250 ppm
	Mean ± S.E.			
Tail Length (µm) F = 32.68; d.f. = 3; *p* < 0.05	3.27 ± 1.32 ^a^	6.85 ± 0.34 ^b^	7.58 ± 0.67 ^c^	14.18 ± 0.28 ^d^
Tail DNA (%) F = 31.23; d.f. = 3; *p* < 0.05	1.13 ± 0.67 ^a^	9.13 ± 0.67 ^b^	13.86 ± 0.23 ^c^	18.23 ± 0.06 ^d^
Tail Movement (µm) F = 51.53; d.f. = 3; *p* < 0.05	0.11 ± 0.13 ^a^	3.26 ± 0.29 ^b^	12.45 ± 0.96 ^c^	14.68 ± 0.56 ^d^
Comet Length (µm) F = 34.33; d.f. = 3; *p* < 0.05	8.32 ± 01.89 ^a^	15.36 ± 0.87 ^b^	17.05 ± 0.49 ^c^	20.62 ± 0.64 ^d^
Head Length (µm) (F = 13.46); d.f. = 3; (*p* < 0.05)	7.03 ± 0.78 ^a^	11.84 ± 0.31 ^b^	15.98 ± 0.39 ^c^	23.75 ± 0.27 ^d^
Head DNA (%) (F = 34.91); d.f. = 3; (*p* < 0.05)	94.72 ± 0.98 ^d^	54.56 ± 0.67 ^c^	46.64 ± 0.86 ^b^	39.19 ± 0.92 ^a^

Where ^a^, ^b^, ^c^, and ^d^ mean different letters within same row are statistically significant according to Tukey’s HSD test. F = ratio of between group variation and within group variation; d.f. = degree of freedom and *p* < 0.05 = statistically significant.

## Data Availability

All data is provided in the manuscript.

## Sample Availability

Sample of the culture can be provided on request.
